# Molecular Prerequisites for Neutrophil Extracellular Trap Formation and Evasion Mechanisms of *Staphylococcus aureus*

**DOI:** 10.3389/fimmu.2022.836278

**Published:** 2022-02-14

**Authors:** Maren von Köckritz-Blickwede, Volker Winstel

**Affiliations:** ^1^ Department of Biochemistry, University of Veterinary Medicine Hannover, Hannover, Germany; ^2^ Research Center for Emerging Infections and Zoonoses (RIZ), University of Veterinary Medicine Hannover, Hannover, Germany; ^3^ Research Group Pathogenesis of Bacterial Infections, TWINCORE, Centre for Experimental and Clinical Infection Research, a joint venture between the Hannover Medical School and the Helmholtz Centre for Infection Research, Hannover, Germany; ^4^ Institute of Medical Microbiology and Hospital Epidemiology, Hannover Medical School, Hannover, Germany

**Keywords:** *Staphylococcus aureus*, immune evasion, neutrophil extracelluar traps, pathogenesis, NETosis

## Abstract

NETosis is a multi-facetted cellular process that promotes the formation of neutrophil extracellular traps (NETs). NETs as web-like structures consist of DNA fibers armed with granular proteins, histones, and microbicidal peptides, thereby exhibiting pathogen-immobilizing and antimicrobial attributes that maximize innate immune defenses against invading microbes. However, clinically relevant pathogens often tolerate entrapment and even take advantage of the remnants of NETs to cause persistent infections in mammalian hosts. Here, we briefly summarize how *Staphylococcus aureus*, a high-priority pathogen and causative agent of fatal diseases in humans as well as animals, catalyzes and concurrently exploits NETs during pathogenesis and recurrent infections. Specifically, we focus on toxigenic and immunomodulatory effector molecules produced by staphylococci that prime NET formation, and further highlight the molecular and underlying principles of suicidal NETosis compared to vital NET-formation by viable neutrophils in response to these stimuli. We also discuss the inflammatory potential of NET-controlled microenvironments, as excessive expulsion of NETs from activated neutrophils provokes local tissue injury and may therefore amplify staphylococcal disease severity in hospitalized or chronically ill patients. Combined with an overview of adaptation and counteracting strategies evolved by *S. aureus* to impede NET-mediated killing, these insights may stimulate biomedical research activities to uncover novel aspects of NET biology at the host-microbe interface.

## Introduction

Polymorphonuclear leukocytes (PMNs or neutrophils) are highly abundant immune cells found in human or animal blood (1). As part of the innate immune response, neutrophils represent crucial effector cells that substantially contribute to immune surveillance and the clearance of microbial infections (1, 2). Of note, neutrophils are recruited in large numbers to infectious foci within minutes following pathogen entry as these cells rapidly sense microbial signatures as well as inflammatory signals released by injured or damaged host tissues (1–3). Specifically, neutrophils express an array of cellular receptors including pattern recognition receptors (PRRs), G protein-coupled receptors (GPCRs), and Fc receptors that mediate recognition of danger signals, thereby governing chemotactic routing and extravasation of PMNs from blood vessel into infected tissues (1, 4). Concurrently, subcellular signaling cascades downstream of these receptors initiate pathogen-eradicating processes that encompass phagocytosis, degranulation, and the biogenesis of reactive oxygen species (ROS) (4). Moreover, neutrophils may kill pathogens by the formation of neutrophil extracellular DNA traps (NETs), an extracellular matrix composed of nuclear and mitochondrial DNA loaded with cell-specific proteases, antimicrobial peptides, and granular proteins (5, 6). This mechanism is of particular importance for controlling infectious diseases as NETs not only display antimicrobial properties, but also exhibit pathogen-capturing features that help to limit the dissemination of microbes in the mammalian host (5, 7). Nevertheless, clinically relevant pathogens have evolved refined counteracting strategies that mediate tolerance or even evasion from extracellular trap-mediated killing (6, 8). A prominent example is *Staphylococcus aureus*, a deadly and multidrug-resistant Gram-positive bacterium that colonizes approximately 30% of the human population (9, 10). This microbe is a very frequent cause of superficial skin and soft tissue infections as well as life-threatening diseases including sepsis, septic arthritis, endocarditis, or pneumonia (10, 11). Notably, *S. aureus* infection triggers a conspicuously strong infiltration of neutrophils into infectious foci which is typically coupled with excessive NET-formation and the development of large abscesses (7, 12, 13). Surprisingly though, the antimicrobial repertoire of NETs often fails to promote clearance of persistent *S. aureus* infections in humans or animal hosts (14–17). On the contrary, *S. aureus* readily outsmarts neutrophil responses and even exploits NET-formation to kill bystander immune cells, highlighting the cunning lifestyle of this microbe (18, 19).

Herein, we briefly summarize the molecular prerequisites of suicidal NETosis, a distinct form of programmed cell death, and vital NET-formation by viable neutrophils in response to various staphylococcal effector molecules. Particularly, we describe how *S. aureus* simultaneously provokes and takes advantage of NET formation during acute and relapsing infections. Further, we review adaptation strategies of staphylococci that confer tolerance to NET-mediated entrapment and killing. Finally, we discuss the pathophysiological potential of NET-controlled infectious foci in the context of severe and chronic *S. aureus* diseases, with the overall aim to stimulate scientific investigations that may lead to the conception of new therapeutic approaches to fight antibiotic-resistant *S. aureus* and other NET-evading pathogens.

## Molecular Mechanisms of Suicidal NETosis and Vital NET Formation

The formation of NETs can be initiated by a variety of stimuli including microorganisms, antibodies, immune complexes, microcrystals, and certain chemicals (6, 20, 21). Various pro-inflammatory cytokines have also been reported to interfere with the release of NETs from PMNs (20, 21). For example, interleukin 1-β (IL-1β), IL-6, IL-8, as well as tumor necrosis factor alpha (TNF-α) elicit NETs and thus may represent crucial immunoregulatory effector molecules of the host that presumably modulate NETosis during specific inflammatory or infectious diseases (5, 20, 22–27).

**Figure 1:**
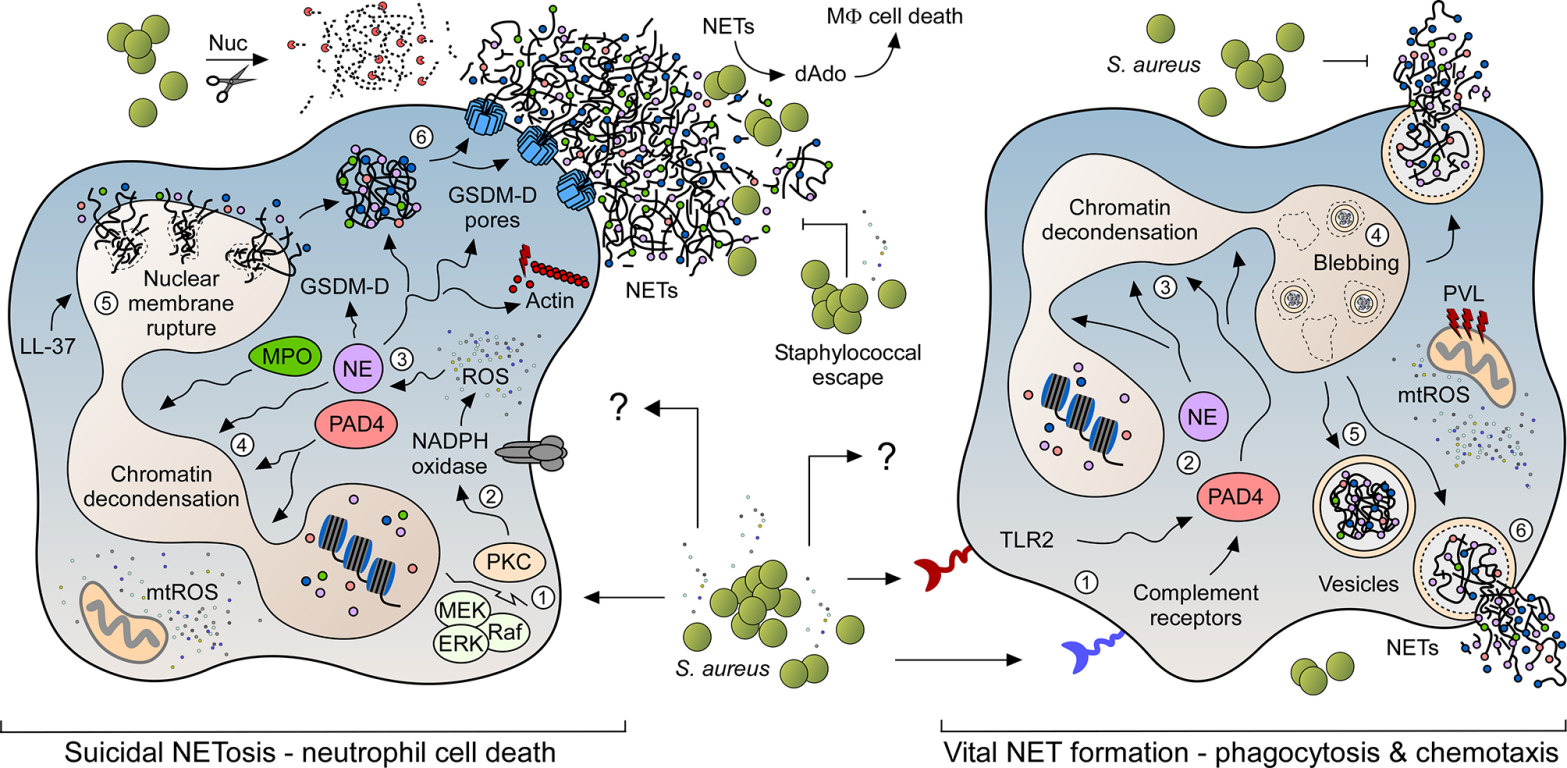
NET formation pathways in response to *S. aureus*. NET formation in response to live staphylococci and their exoproducts may occur *via* two predominant signaling pathways. While suicidal NETosis leads to neutrophil cell death (left panel), rapidly occurring vital NET formation retains the ability of PMNs to migrate and phagocytose bacterial invaders (right panel). Numbers 1-6 in each panel indicate the order of events during NET formation. *S. aureus* readily escapes from NET-mediated entrapment and killing by secreting multiple virulence determinants (e.g. thermonuclease (Nuc)), thereby boosting staphylococcal persistence and dissemination of disease. Characteristic features and key host signaling molecules including toll-like receptor 2 (TLR2), myeloperoxidase (MPO), neutrophil elastase (NE), protein-arginine deiminase type 4 (PAD4), cathelicidin LL-37, reactive or mitochondrial reactive oxygen species (ROS; mtROS), protein kinase C (PKC), Raf–MEK–ERK cascade (Raf; MEK; ERK), membrane-bound NADPH oxidase, processed gasdermin D (GSDM-D) and associated pores are highlighted.

To date, two predominant pathways have been described in detail that lead to the expulsion of NETs from neutrophils: suicidal (lytic) NETosis, which causes neutrophil cell death, and vital NET formation that results in the release of NET-loaded vesicles from viable PMNs (**Figure 1**) (21, 28). Suicidal NETosis, a process that provokes NET formation within 3-4 hours post-stimulation (23), largely depends on calcium signaling along with the protein kinase C (PKC)- or Raf-MEK-ERK pathway-dependent activation of the membrane-bound NADPH oxidase, which represents a multisubunit protein complex that synthesizes ROS for the subsequent activation of PAD4 (protein-arginine deiminase type 4) (**Figure 1**) (6, 21, 23, 29, 30). PAD4 is a Ca^2+^-binding protein and key driver of NETosis as it causes citrullination of core histone (6, 31). Particularly, activated PAD4 migrates to the nucleus where it catalyzes the conversion of positively charged arginine residues of histones into citrullines, thereby inducing the process of chromatin decondensation (6, 31). Concurrently, ROS break down the azurosome, a serine protease- as well as myeloperoxidase (MPO)-encompassing protein complex found in neutrophil granules (32). In this manner, azurophilic proteases (e.g. azurocidin, cathepsin G, and neutrophil elastase (NE)) as well as MPO are released into the cytosol, where NE disrupts the actin cytoskeleton and simultaneously traffics together with MPO to the nucleus, further contributing to histone degradation and chromatin decondensation (6, 32, 33). Interestingly, recent work demonstrated that mitochondrial ROS (mtROS) can also contribute to the phenotype of suicidal NETosis, particularly in response to *S. aureus* (34, 35). Subsequent steps involve disruption of the nuclear cell envelope and electrostatic interaction of core-derived decondensed chromatin with cytosolic and granular proteins in the cytoplasm (**Figure 1**) (23). In this scenario, the human cathelicidin LL-37 and its mouse analogue mCRAMP (mouse cathelicidin related antimicrobial peptide) have been shown to contribute to the perforation of nuclear membranes in NET-forming neutrophils (36). Moreover, emerging literature suggests that NE-processed gasdermin D (GSDM-D), a crucial executor protein of the pyroptotic cell death pathway (37–39), may also alter the membrane integrity of PMNs at this stage (30, 40, 41). Specifically, cleaved GSDM-D displays potent pore-forming and membrane-damaging capacities and is therefore believed to puncture the core as well as the plasma membranes of netting neutrophils, ultimately culminating in the expulsion of antimicrobial NETs from dying PMNs into the extracellular space (**Figure 1**) (30, 37–41). Of note, processed GSDM-D may further contribute to a non-canonical form of suicidal NETosis, which occurs exquisitely in response to intracellular Gram-negative bacteria (42). In summary, suicidal NETosis of neutrophils differs from other cell death mechanisms based on the specific phenomenon that chromatin decondensation and disintegration of the nuclear membrane occurs concomitant with cytoplasmic granule dissolution, allowing the NET components to mix in the cytoplasm prior to their extracellular release (23).

NET formation can also be induced *via* a secondary mechanism which retains PMN integrity and viability by an active release of DNA-containing vesicles (**Figure 1**). This process (i.e. vital NET formation; sometimes referred to as vesicular NETosis) is mainly NADPH oxidase-independent and rapidly occurs within less than hour upon stimulation (43, 44). Activation of vital NET formation involves complement-mediated pathogen opsonization or sensing *via* toll-like receptors (TLRs) (44). Particularly, Clark and colleagues have initially shown that TLR-4-activated platelets trigger vital NET formation in order to capture bacteria in septic blood (45), while subsequent *in vitro* and *in vivo* work by the same laboratory demonstrated vital NET release and involvement of TLR-2 and complement receptor 3 upon neutrophil stimulation with Gram-positive bacteria (7, 43). Similar to suicidal NETosis, vital NET formation has also been shown to be partially dependent on calcium influx and activated PAD4 and NE, both of which migrate to the nucleus to initiate unpacking of histones and chromatin decondensation (**Figure 1**) (28, 46). Subsequently, nuclear envelope blebbing leads to the formation of DNA-containing vesicles that eventually fuse with the plasma membrane to expel their antimicrobial content into the extracellular space (**Figure 1**) (44). Notably, this process does not damage the plasma membrane so that neutrophils initially maintain their ability to migrate and phagocytose microbial invaders (7, 43). At later stages, however, the nuclear membrane may rupture thereby causing an accumulation of chromatin fibers in the cytosol (43). Thus, vital NET formation may indirectly be coupled with suicidal NETosis and lytic NET formation respectively. In this regard, we finally note that a specific form of vital NET formation involves a rapid release of mitochondrial DNA (mtDNA) from viable PMNs (47). Opposed to the canonical pathway of vital NET formation, this form of vital NET release requires the activity of the NADPH oxidase along with granulocyte-macrophage colony-stimulating factor (GM-CSF)-mediated priming of neutrophils, followed by subsequent stimulation by complement factor 5a (C5a) or lipopolysaccharide (LPS) (47). Yet, mechanistic details and the exact role of this form of vital NET release during infectious diseases or other pathophysiological conditions remain largely unknown.

## Staphylococcal Catalysts of NET Formation

**Figure 2:**
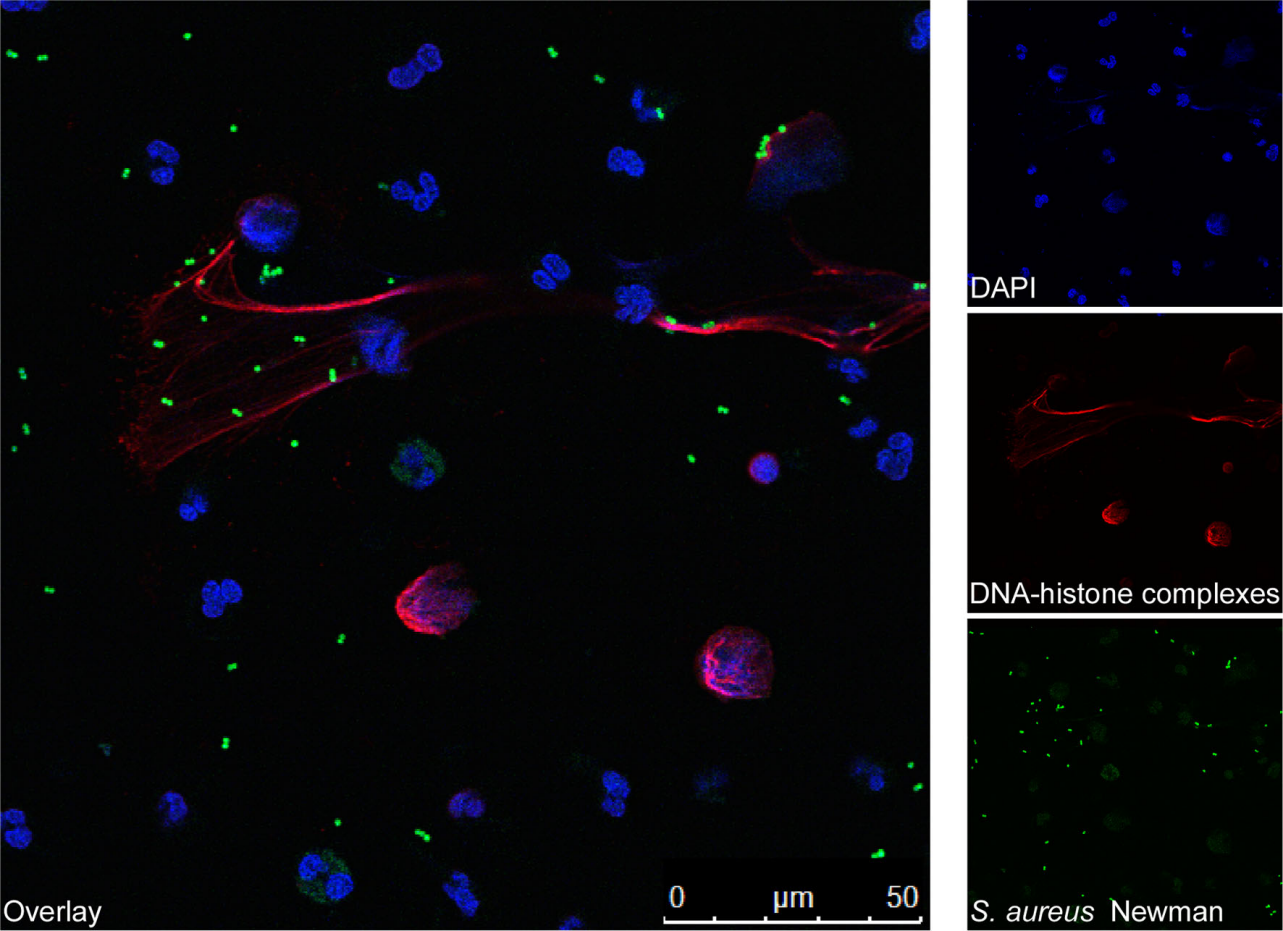
Fluorescence microscopy images of human blood-derived neutrophils forming NETs during incubation with *S. aureus.* Human blood-derived neutrophils were stimulated with FITC-labelled *S. aureus* Newman for 90 min (green). Next, the formation of NETs was visualized using confocal fluorescence microscopy with antibodies against DNA-histone-complexes (red) as previously described (48). Nuclei were stained with DAPI (blue). The main image shows staphylococci (green) entrapped by NETs (red).

Neutrophils release NETs in response to multiple infectious agents such as Gram-negative and Gram-positive bacteria (20, 43). Intriguingly, *S. aureus* is considered one of the most potent inducers of NETosis, irrespective of whether PMNs sense live or metabolically inactive (dead) staphylococci (43). Nevertheless, the magnitude of NET induction is significantly increased when PMNs are exposed to viable *S. aureus* cells or staphylococcal culture supernatants, suggesting that secreted exoproteins together with intact structural components of the bacterial cell envelope substantially affect *S. aureus*-triggered NET formation (**Figure 2**) (43). In fact, various studies revealed that stimulation with live bacteria or *S. aureus* pore-forming toxins contribute to this phenomenon (**Table 1**) (23, 34, 43, 51, 56). For example, the staphylococcal bi-component toxin PVL (Panton-Valentine leukocidin) has been shown to prime vital NET formation in freshly isolated human neutrophils (43, 56). In this NADPH-independent process, endocytosed PVL particularly targets mitochondria thereby triggering the formation of mtROS from these organelles (**Figure 1**) (56). Further, PVL-mediated release of NETs involves MPO, Ca^2+^-signaling and PAD4 activation, as well as citrullination of histone H3 (56). However, PVL-induced NET formation seems to be highly dose-dependent as elevated levels of PVL may promote necroptotic or apoptotic cell death rather than classical NETosis (61, 62). Dose-dependency may also play a crucial role during NET formation induced by LukAB (also known as LukGH), another staphylococcal pore-forming toxin that lineage-dependently targets human CD11b or the hydrogen voltage-gated channel 1 (HVCN1) (51, 63, 64). Like PVL, LukAB consists of two subunits and promotes NET release from PMNs exclusively at sublytic concentrations *in vitro* (**Table 1**) (51). LukAB-induced NETs, however, did not display enhanced bactericidal activity towards staphylococci suggesting that *S. aureus* may systematically induce or even exploit NET formation during persistent infections (51). In agreement with this idea, recent studies by Bhattacharya et al. uncovered that extracellular traps augment chronic staphylococcal infections (15). Specifically, the combined activity of LukAB and PVL provokes NET formation within biofilms, a rather detrimental effect that affects the clinical outcome of chronic burn wounds in pigs (15). In this regard, we note that many staphylococcal toxins and virulence determinants are controlled by the global accessory regulatory system Agr (65). Agr is a quorum sensing system that consists of several structural components including AgrD, the precursor molecule of the autoinducing peptide AIP which facilitates staphylococcal communication and target gene regulation *via* activation of AgrC-AgrA two component system (65). Surprisingly, truncated and formylated peptide variants of AgrD solely exhibit immunomodulatory and NETosis-promoting capacities, a phenomenon which raises the question of whether staphylococci directly process components of the Agr system to enhance NET formation in infectious foci (**Table 1**) (50). Agr is also controlling an array of amphipathic, α-helical peptides designated phenol-soluble modulins (PSMs) (65, 66). These small peptides have strong lytic properties and usually trigger necroptotic or pyroptotic cell death in target cells (66, 67). At micromolar concentrations, purified PSM-α peptides were also found to initiate a very rapidly occurring and NADPH oxidase-independent form of NETosis in purified PMNs, a process that is reminiscent of vital NET formation (57). Moreover, PSM-α peptides govern budding of lipoprotein-containing extracellular membrane vesicles from staphylococcal cytoplasmic membranes (68, 69). Since *S. aureus*-derived lipoproteins constitute potent inducers of TLR-2 (70–72), and TLR-2 signaling along with complement-mediated opsonization correlates with vital NETosis in human neutrophils (7), lipoprotein-comprising plasma membrane preparations of the methicillin-resistant *S. aureus* (MRSA) strain USA300 have recently been identified as another potent driver of vital and PAD4-depenent NET formation (**Table 1**) (52).

**Table 1:** Selected staphylococcal factors that induce or interfere with NET formation.

Staphylococcal factor	Gene	Category	Effect	References
Adenosine synthase A	*adsA*	Surface protein	interfering factor; collaborates with Nuc to convert NETs into cytotoxic dAdo thereby preventing macrophage infiltration into abscesses	(17, 49)
AgrD	*agrD*	Precursor molecule of the autoinducing peptide AIP	promotes NET formation in human neutrophils^1^	(50)
Autolysin	*atl*	Peptidoglycan hydrolase	indirect effector molecule of vital NET formation	(43)
Leukocidin AB	*lukAB*	Pore-forming toxin	triggers NET formation^2^; interacts with PVL to promote release of NETs in biofilms	(15, 51)
Lipase	*geh*	Exoenzyme	putative role during vital NETosis	(43)
Lipoproteins	—^3^	Plasma membrane components	potent driver of vital and PAD4-depenent NET formation	(52)
Extracellular adherence protein	*eap*	Exoprotein^4^	interfering factor; disturbs the stability of the NET scaffold by binding and aggregating DNA fibers; exhibits neutrophil serine proteases-blocking capacities	(53, 54)
Extracellular adherence protein homolog 1	*eapH1*	Exoprotein^4^	interfering factor; inhibits neutrophil serine proteases	(54)
Extracellular adherence protein homolog 2	*eapH2*	Exoprotein^4^	interfering factor; blocks neutrophil serine proteases	(54)
Fibronectin-binding protein B	*fnbB*	Surface protein	neutralizing factor; scavenges NET-associated histones	(55)
Panton-Valentine leukocidin	*lukFS*	Pore-forming toxin	primes vital NET formation in human PMNs^2^; collaborates with LukAB to drive NET release within biofilms; punctures mitochondria to induce alternative NETosis	(15, 43, 56)
Phenol-soluble modulins (α-type)	*psmα1-4*	Cytolysin	initiates an NADPH oxidase-independent and vital-like form of NETosis	(57)
Staphylococcal protein A	*spa*	Surface protein	stimulates formation of NETs from human PMNs^5^	(58)
Second binding protein of immunoglobulin	*sbi*	Surface protein	triggers together with protein A the release of NETs^5^	(58)
Thermonuclease	*nuc*	Exoenzyme	neutralizing factor; degrades NETs during local, systemic, and chronic *S. aureus* infection thereby catalyzing escape from NET-mediated entrapment and killing; converts NETs together with AdsA into dAdo to promote persistent infections	(14, 16, 17, 34, 59)

^1^NETosis induction requires truncated peptide variant of AgrD.

^2^occurs at sublytic concentrations.

^3^no specific gene listed as S. aureus genomes bear approximately 70 lipoprotein-encoding genes (60).

^4^member of the SERAM (secretable expanded repertoire adhesive molecules) protein family.

^5^mechanism requires live staphylococci and probably an additional secreted cofactor.

Apart from toxin- or lipoprotein-mediated induction of NET formation, certain peptidoglycan-associated molecules synthesized by staphylococci contribute to the release of extracellular traps from PMNs (**Table 1**). For example, staphylococcal protein A (SpA) and Sbi (second binding protein of immunoglobulin), both cell surface-displayed proteins that exhibit strong immunoglobulin-binding properties (73), affect the generation of NETs as a *S. aureus* Newman Δ*spa* Δ*sbi* double-mutant lost the ability to promote release of NETs from human neutrophils in tissue culture-based assays (58). In this context, Hoppenbrouwers et al. discovered that the amount of SpA in staphylococcal cell walls correlates with the capacity of *S. aureus* to provoke NETosis (58). Isolates that display elevated amounts of SpA in the cell wall (SpA^high^) activated NET formation more efficiently as compared to strains that exhibit a SpA^low^ phenotype (58). However, purified SpA alone failed to stimulate NETosis in purified PMNs indicating that other, presumably secreted factors of live staphylococci along with a unique mechanism are required to promote SpA-associated NET formation (58). This may also hold true for the major autolysin Atl, which has been identified as another possible effector molecule of vital NET formation (43). Albeit release of NETs from human PMNs occurred in response to high concentrations of recombinant autolysin, Atl may rather contribute indirectly to vital NETosis (43). Specifically, Wang et al. demonstrated that the peptidoglycan-hydrolyzing activity of Atl and Sle1, an N-acetylmuramyl-L-alanine amidase (74), impacts trafficking of *S. aureus*-derived membrane vesicles across murein layers (68), and thus may contribute to lipoprotein-induced stimulation of TLR-2 and vital NET formation. Finally, we note that a staphylococcal lipase has also been implicated in vital NETosis, although mechanistic details remain elusive (43). Together, these comprehensive studies demonstrate that *S. aureus* together with its exoproducts predominantly elicits vital NET formation, albeit viable staphylococci may also skew neutrophils toward suicidal NETosis in a manner requiring NADPH oxidase-derived ROS or even mtROS, especially when co-cultivated with PMNs for longer time periods or during infection of the heart (**Figure 1**) (23, 34, 35).

## Staphylococcal Evasion From NET-Mediated Entrapment and Killing

Pioneering work by Brinkmann et al. uncovered that NETs have pathogen-capturing and antimicrobial properties (5). Multiple microbes including bacteria, viruses, and fungi can be entangled, disarmed, or neutralized by these extracellular structures, readily preventing pathogen spread and dissemination of disease (5, 27, 44). Of note, not only various NET-associated factors such as calprotectin, histones, cathepsin G, or the DNA itself have been discussed to mediate antimicrobial activity of NETs (5, 75–77), but also the boosting of NET-formation has been demonstrated to be protective in some disease conditions, e.g. during systemic *S. aureus* infections in mitochondrial calcium uptake 1 (MICU1)-deficient (*MICU1* ^-/-^) mice or during lung infections upon treatment with statins that block cholesterol biosynthesis (35, 78). However, NETs often fail to eradicate replicating *S. aureus* during persistent infections as this pathogen releases a plethora of virulence factors into the extracellular milieu that antagonize NET-mediated entrapment and killing (**Table 1**) (79). For example, *S. aureus* secretes a robust thermonuclease (Nuc) which rapidly dismantles NETs thereby affecting local, systemic, as well as chronic infections (**Figure 1**) (14, 16, 17, 34, 59). In that regard, we note that Nuc-mediated degradation of NETs may further restrict the communication of PMNs and macrophages (34). This striking observation seems to be of particular importance during slowly-occurring suicidal NETosis since an accelerated and mtROS-controlled form of lytic NETosis, as observed in S100A9-deficient neutrophils, abrogated Nuc-derived effects and simultaneously enhanced macrophage-mediated killing of *S. aureus* and other extracellular bacterial pathogens (34). Nonetheless, macrophages often reside at the periphery of infectious foci in wild-type animals, a pathophysiological phenomenon that also involves the activity of Nuc (17). Particularly, Nuc-mediated degradation of NETs together with the activity of AdsA (adenosine synthase A), a cell wall anchored 5’-3’-nucleotidase, leads to the biosynthesis of deoxyadenosine (dAdo) (**Figure 1**) (17). dAdo is a cytotoxic deoxyribonucleoside which exquisitely kills macrophages during abscess formation by targeting the purine salvage pathway and apoptotic signaling cascade (17, 49, 80). In this manner, *S. aureus* and related staphylococci not only prevent NET-mediated killing within abscesses, but rather exploit excretion of NETs to suppress phagocyte entry into deeper cavities of infectious foci, ultimately enhancing pathogen survival and establishment of persistent infections (17, 49, 81). Overall, staphylococcal Nuc represents a key determinant utilized by staphylococci to prevent NET-associated enmeshment and killing.

*S. aureus* evolved additional virulence determinants to block NET-mediated entrapment (**Table 1**). Most of these Nuc-independent mechanisms target the structural backbone of NETs along with NET-associated proteins such as histones, NE, or cathepsin G (53–55). Staphylococcal extracellular adherence protein (Eap), for example, binds and aggregates NET fibers *in vitro* and therefore affects the formation and stability of neutrophil-derived DNA traps (53). Together with two orphan Eap homologes (EapH1 and EapH2), Eap has also neutrophil serine proteases-blocking capacities, as proteins of the Eap family efficiently bind to the catalytic domains of NE, cathepsin G, or proteinase 3 (54). Accordingly, *S. aureus* variants lacking the Eap protein-encoding genes are attenuated in a mouse model of blood stream infection (54). More recent *in vitro* work showed that staphylococcal fibronectin-binding protein B (FnBPB) contributes to the neutralization of NETs (55). In this model, FnBPB binds with very high affinity to histones thereby suppressing their bactericidal activity (55). Scavenging histones further enhances survival of live *S. aureus* exposed to NETs since FnBPB-deficient mutant cells were more prone to NET-mediated killing as compared to the enmeshed parental *S. aureus* strain (55). Finally, we note that *S. aureus* alters the net charge of the bacterial cell surface by lysinylating membrane phosphatidylglycerol and alanylating teichoic acids (TAs) (82, 83). Interestingly, D-alanylation of TAs by the DltABCD machinery together with the formation of a capsule mediates tolerance to the microbicidal attributes of NETs in *Streptococcus pneumoniae* (84), raising the possibility that alanylated TAs and encapsulation may similarly protect *S. aureus* from NET-mediated killing. In this context, it should also be considered that D-alanylation of TAs confers resistance to several cationic antimicrobial peptides including the NET-associated cathelicidin LL-37 (82, 85). However, Jann et al. uncovered that *S. aureus* wild type bacteria and their *dltA* variants were killed by NETs in a similar fashion, indicating that neutrophils use the cathelicidins mainly for the phagolysosomal but not NET-associated antimicrobial defense (86). In agreement with this data, previous *in vitro* work found that particularly LL-37 lost its antimicrobial properties when bound to NETs, and rather represents a stabilization factor that antagonizes Nuc-mediated degradation of extracellular traps (48). This may also explain why *S. aureus* secretes further nucleases including the membrane-bound Nuc2 enzyme as well as EssD (or EsaD), a substrate of the staphylococcal type VII secretion apparatus (87–89). Direct evidence that these enzymes significantly contribute to the escape from NET-mediated entrapment has, however, not been provided so far. Collectively, *S. aureus* secretes multiple virulence factors to incapacitate the microbicidal features of NETs, presumably to promote immune evasion and dissemination in the mammalian host.

## Immunopathological Consequences of Aberrant NET Formation During Staphylococcal Infections

Recent advances suggest that excessive or dysregulated NETosis may cause severe pathologies during various human diseases (6, 90). During acute or chronic infections, for example, aberrant NET formation has been linked to inflammatory processes, tissue and organ injury, and negative disease outcomes along with increased mortality rates (6, 91). This is exemplified in the context of pulmonary diseases and cystic fibrosis (CF), a genetic disorder caused by a mutation in the CF transmembrane conductance regulator-encoding gene *CFTR* (91, 92). CF patients develop a highly viscous mucus which favors pathogen colonization and infection, especially by NETosis-triggering microbes such as *Pseudomonas aeruginosa* or *S. aureus* (92, 93). Not surprisingly though that sputum samples derived of *P. aeruginosa*- or *S. aureus*-infected CF patients contain elevated amounts of NETs together with NET-bound peptides (16, 94). Yet, NET-rich microenvironments of CF lungs that are often associated with hypoxia (95), a condition that retains the ability of PMNs to form NETs in response to staphylococci (96), typically fail to clear microbial invaders and rather initiate detrimental events that affect lung pathology and chronic airway inflammation (16, 91). Particularly, several NET-associated proteins exhibit cytotoxic and tissue destructive capacities (97–100). For example, sputum specimens from CF patients were found to contain large amounts of MPO along with MPO-derived oxidizing and nitrating species (94, 100). These factors may contribute to respiratory dysfunction and poor disease prognosis in *S. aureus*-infected CF patients, as MPO and MPO-derived oxidants confer tracheobronchial or alveolar epithelial cell damage (99, 100). Epithelial injury and NET-associated cytotoxicity toward CF lung tissue may further occur in response to NET-assembled histones or NE that have also been identified in sputum samples of affected individuals (94, 101). Especially extracellular histones display toxigenic properties toward epithelial and endothelial cells due to their membrane-binding and damaging attributes (97, 99). Similarly, uncontrolled release of NE and NE-DNA complexes during MRSA-induced pneumonia is considered as a substantial mediator of acute lung injury and may therefore exacerbate disease outcomes of staphylococcal pulmonary infections (**Table 2**) (98, 113). In light of this, increased concentrations of histones, NE, and calprotectin, a NET-bound alarmin (75), could potentiate the release of cytokines and chemokines into lung fluids (102, 116, 117), presumably explaining hyper-inflammatory responses and the non-ending recruitment of neutrophils along with excessive NETosis in *S. aureus*-infected CF lungs. Thus, dysregulated NETosis potentially mediates adverse and harmful effects during CF and *S. aureus*-caused infections of the respiratory tract (**Table 2**).

**Table 2:** Detrimental effects of NETs during infection with *S. aureus*.

Type of infection or medical condition	Consequence of NET formation or aberrant NETosis^1^	References
Abscess	NETs trigger staphylococcal persistence and macrophage cell death as a result of Nuc- and AdsA-mediated conversion of these structures into cytotoxic dAdo	(17, 49)
Burn wound	toxin-induced release of NETs tunes survival of MRSA within chronic burn wounds and biofilms in pigs	(15)
Cystic fibrosis	NETs represent a key source of inflammation and presumably affect staphylococcal long-term persistence in cystic fibrosis lungs^2^	(16, 102, 103)
Diabetes	NET-overproduction by low-density neutrophils increases susceptibility of diabetic mice to *S. aureus* blood stream infection; NETs impair wound healing in diabetics, probably complicating staphylococcal skin and deep tissue infections^2^	(104, 105)
Infective endocarditis	NETs facilitate *S. aureus* vegetation formation on damaged heart valves in an experimental endocarditis rat model	(106)
Inflamed skin	enhanced NET formation at injured body sites promotes *S. aureus* skin colonization in mice	(107)
Psoriasis	NETs potentially correlate with increased *S. aureus* colonization rates in psoriatic patients^2^	(108–112)
Pneumonia	abnormal NETosis in response to MRSA provokes lung injury in a mouse model of acute respiratory infection	(113)
Sepsis	excessive release of NETs from neutrophils triggers intravascular coagulation and tissue injury in septic mice	(114, 115)

^1^observed in laboratory animals as indicated; ^2^putative effects.

Dysfunctional NETosis can also complicate staphylococcal infections in the context of other chronic diseases (**Table 2**). In diabetic mice, staphylococcal α-toxin drives the transforming growth factor β (TGF-β)-signaling-dependent expansion of low-density neutrophils (LDN) (104). LDN in turn excrete large amounts of NETs, an adverse feature that has been linked to increased mortality rates in mice challenged with the community-acquired MRSA strain USA300 (104). These effects may clarify why diabetic patients are more vulnerable to *S. aureus* bacteremia as compared to non-diabetic individuals (118). In this regard, we further note that NETs alter the wound healing process in patients with diabetes (105), probably explaining why this population often suffers from complicated *S. aureus*-mediated skin or foot ulcer infections (119). Moreover, recent work by Bitschar et al. revealed that NETs interact with keratinocytes at injured or inflamed skin sites thereby promoting *S. aureus* vegetation formation on body sites that are typically not colonized by this microbe (107). This appears highly relevant as *S. aureus* is frequently isolated from the skin of patients with atopic dermatitis or psoriasis (108–110, 120), both chronic inflammatory skin diseases that affect large segments of the human population (121, 122). At least skin lesion sites of psoriatic patients are characterized by increased amounts of NETs (111, 112), which could correlate with increased *S. aureus* colonization rates. Nonetheless, aberrant NETosis can also affect staphylococcal disease pathogenesis in otherwise healthy individuals and immunocompetent laboratory animals as, for example, enhanced neutrophil influx and NET formation boost biofilm and implant-associated infections in wild-type mice (15, 123). Further, it is worth noting that a massive release of NETs in foci of infection causes tissue damage and organ injury during staphylococcal systemic infection (**Table 2**) (114). Innovative *in vivo* imaging technologies uncovered that NETs along with NET-bound peptides accumulated in the liver vasculature of septic mice (114). Here, NET-associated NE and histones were found to co-localize with necrotic tissue sites suggesting that NET components exhibit organ-damaging attributes during severe staphylococcal diseases (114). In line with these observations, McDonald and colleagues discovered that a sepsis-provoked release of NETs into the vasculature triggers networking of platelets and extracellular traps, ultimately leading to intravascular coagulation and injury of hepatic tissues (115). Finally, the platelet-NET axis impairs *S. aureus*-induced infective endocarditis (106). Specifically, NETs have been found to amplify *S. aureus* vegetation formation on injured heart valves in an experimental rat model of infective endocarditis (106), a fatal side-effect of NETs that is also exploited by other endocarditis-promoting pathogens such as *Streptococcus mutans* (124). Collectively, these compelling studies demonstrate that excessive formation of NETs along with elevated levels of NET-associated peptides in response to acute or chronic staphylococcal infections can be detrimental to the mammalian host, particularly in the context of systemic or pulmonary diseases.

## Concluding Remarks

NETs as part of the innate immune response are generally believed to correlate with clinical outcomes of many infectious diseases. As long as the magnitude of NET formation is coordinated and tightly balanced, these extracellular structures exhibit beneficial properties and contribute to the entrapment, disarming, and killing of microorganisms (5, 6). This may also hold true for local infections caused by *S. aureus* as NETs are not only formed within abscesses but also diminish the risk of pathogen entry into circulating body fluids and the development of invasive diseases (7). Paradoxically, NETs may exacerbate staphylococcal infections and disease progression, specifically when excessively synthesized during acute or chronic infections (16, 104, 106, 114, 115). This raises the question of whether *S. aureus* selectively induces or even gains advantage of NET formation under certain pathophysiological conditions. In line with this model, earlier work demonstrated that induction of NETosis promotes intra-abscess survival of *S. aureus*, colonization of injured skin sites, and biofilm formation (15, 17, 107). As aberrant NETosis is also linked to severe staphylococcal infections and the establishment of pulmonary infections in chronically ill patients (16, 114), it is further tempting to speculate that *S. aureus* may take advantage of the organ-damaging capacities of NETs or NET-associated components to traverse endothelial or epithelial barriers for subsequent penetration of deep tissues. If so, concurrent stimulation and exploitation of excessive NETosis may represent a refined immune-evasive maneuver evolved by *S. aureus* to create new proliferative niches in the mammalian host, a fact that may clarify why staphylococci excrete a plethora of NET-inducing effector molecules into the extracellular space. In this context, it should also be taken into account that NETosis-catalyzing molecules released by *S. aureus* represent predominant immune evasion molecules, most of which the pathogen secretes in any way to manipulate or kill host cells (19, 125, 126). This is exemplified by staphylococcal LukAB which primarily lyses various immune cells but concurrently has the capacity to trigger NET formation in human neutrophils (51, 127). Expulsion of microbicidal NETs from viable or dying neutrophils may therefore simply reflect an inadvertent side-effect within foci of infection that *S. aureus* readily tolerates or even exploits due to the biogenesis of numerous virulence and entrapment-protective factors such as Nuc. Nevertheless, owing to the expression of these evasion molecules, it appears irrelevant at first glance whether the host induces vital or suicidal NETosis during *S. aureus*-mediated infections. When compared to suicidal NETosis, however, a rapid release of vital NETs might be more advantageous for mammalian hosts in terms of combating staphylococci as this route of NET generation not only mediates prompt trapping of the microbe at very early infection stages, but also retains the ability of PMNs to crawl and phagocytose the bacterial invader (7, 43). Further, vital NET formation is known to maintain the membrane integrity of netting PMNs (7, 43) and thus presumably evolved to limit a release of inflammation-promoting and otherwise-sequestered intracellular molecules, ultimately minimizing the risk of developing a hyper-inflammatory milieu that eventually potentiates tissue injury and staphylococcal disease severity. However, it remains elusive whether *S. aureus*-infected hosts are capable of selectively activating a specific type of NET formation during infection. Assuming a particular form of NETosis is indeed more effective against *S. aureus* but concomitantly less detrimental to the host, the discovery of appropriate host factors controlling specific NET-forming events along with the optimization of individual therapeutics or antibiotics, some of which are known to interfere with NETosis or NET-mediated killing of MRSA (128–131), may help to better manage staphylococcal infectious diseases in the future.

Overall, the formation of NETs shapes staphylococcal disease pathogenesis and clinical manifestations in many aspects. Whilst NETs display antimicrobial properties and to some extend reduce pathogen spread, these web-like matrices may also unfold adverse characteristics and constitute a bio-scaffold utilized by staphylococci to establish persistent infections in humans or animal hosts. Thus, deciphering all molecular facets and mechanistic details by which clinical *S. aureus* isolates stimulate or manipulate various forms of NET formation, along with the discovery of contributing host signaling cascades and NET-stabilizing factors, may help to conceive innovative and selective therapeutic approaches to improve staphylococcal infection outcomes, especially in hospitalized or critically ill patients.

## Ethics Statement

Blood samples were drawn from healthy donors in agreement with the local ethical board. The study was approved by the medical ethics committee of the Hannover Medical School (Hannover, Germany) under the permission number 3295-2016.

## Author Contributions

VW performed the literature review as well as data collection and prepared the manuscript draft. MK-B provided revisions, additional literature review, comments, and essential materials. Both authors substantially contributed to the article and approved the submitted version.

## Funding

We are grateful for support from the German Research Foundation (project grant WI4582/2-1 to VW, project number 449712894; project grant KO 3552/4-1 to MK-B) and from Else Kröner-Fresenius-Stiftung (award 2021_EKEA.16 to VW). Furthermore, this work was partially supported by the R2N project under grant (74ZN1574) provided to MK-B, which is funded by the Federal State of Lower Saxony.

## Conflict of Interest

The authors declare that the research was conducted in the absence of any commercial or financial relationships that could be construed as a potential conflict of interest.

## Publisher’s Note

All claims expressed in this article are solely those of the authors and do not necessarily represent those of their affiliated organizations, or those of the publisher, the editors and the reviewers. Any product that may be evaluated in this article, or claim that may be made by its manufacturer, is not guaranteed or endorsed by the publisher.
